# MMP-9 Downregulation with Lipid Nanoparticles for Inhibiting Corneal Neovascularization by Gene Silencing

**DOI:** 10.3390/nano9040631

**Published:** 2019-04-18

**Authors:** Josune Torrecilla, Itziar Gómez-Aguado, Mónica Vicente-Pascual, Ana del Pozo-Rodríguez, María Ángeles Solinís, Alicia Rodríguez-Gascón

**Affiliations:** Pharmacokinetic, Nanotechnology & Gene Therapy Group (PharmaNanoGene), Faculty of Pharmacy, Centro de investigación Lascaray ikergunea, University of the Basque Country UPV/EHU, Paseo de la Universidad 7, 01015 Vitoria-Gasteiz, Spain; josune.torrecilla@ehu.eus (J.T.); itziar.gomez@ehu.eus (I.G.-A.); monica.vicente@ehu.eus (M.V.-P.); ana.delpozo@ehu.eus (A.d.P.-R.)

**Keywords:** gene therapy, solid lipid nanoparticles, MMP-9, corneal inflammation, HCE-2 cells, capillary tube formation, RNAi, shRNA

## Abstract

Gene silencing targeting proangiogenic factors have been shown to be a useful strategy in the treatment of corneal neovascularization (CNV). Among interference RNA (RNAi) molecules, short-hairpin RNA (shRNA) is a plasmid-coded RNA able to down-regulate the expression of the desired gene. It is continuously produced in the host cell, inducing a durable gene silencing effect. The aim of this work was to develop a solid lipid nanoparticle (SLN)-based shRNA delivery system to downregulate metalloproteinase 9 (MMP-9), a proangiogenic factor, in corneal cells for the treatment of CNV associated with inflammation. The nanovectors were prepared using a solvent emulsification-evaporation technique, and after physicochemical evaluation, they were evaluated in different culture cell models. Transfection efficacy, cell internalization, cell viability, the effect on MMP-9 expression, and cell migration were evaluated in human corneal epithelial cells (HCE-2). The inhibition of tube formation using human umbilical vein endothelial cells (HUVEC) was also assayed. The non-viral vectors based on SLN were able to downregulate the MMP-9 expression in HCE-2 cells via gene silencing, and, consequently, to inhibit cell migration and tube formation. These results demonstrate the potential of lipid nanoparticles as gene delivery systems for the treatment of CNV-associated inflammation by RNAi technology.

## 1. Introduction

Corneal inflammation, or keratitis, may be caused by many underlying illnesses, including dry eye, eyelid injuries, physical and chemical traumas, and microbial infections (the major cause of corneal inflammation development) [1]. In developing countries, herpes simplex virus type I is the leading cause of keratitis-associated infectious blindness [2]. Common symptoms of keratitis are local pain, tearing, photophobia, blurry vision and ocular redness. When keratitis becomes chronic, visual disturbances occur and often results in tissue damage that leads to corneal ulceration, corneal wound healing, and even perforation, causing visual impairment and blindness [3].

Matrix metalloproteinases (MMPs) are a group of enzymes with a key role in the different phases of the corneal inflammation, from the onset of the epithelial defect to its resolution [4,5]. Among the enzymes included in this group, MMP-9 is one of the primary extracellular matrix remodeling enzymes that participate in pathological conditions of the cornea, including corneal neovascularization (CNV) [6]. Although neovascularization is part of the repair process of extensive damage to the eye surface [7], it can lead to compromised visual acuity because the cornea loses its avascularity feature. Current treatment options for CNV include the topical application of nonsteroidal or corticosteroidal anti-inflammatory agents, immunosuppressive drugs or surgical interventions such as photodynamic therapy cauterization and β-irradiations [8]. However, the limited efficacy and adverse effects restrict the current treatment therapies [9]. For instance, the rise of intraocular pressure is a complication of topical corticosteroid applications with drugs such as dexamethasone or prednisolone [10] while long-term treatment with immunosuppressants increases the risk of infection and some blood diseases [11]. Therefore, the development of alternative strategies becomes necessary, gene therapy being a hot issue of modern medical research work. The treatment of CNV based on gene therapy can be tackled by two different approaches: gene supplementation to express an antiangiogenic factor, and gene suppression to inhibit the synthesis of a proangiogenic factor. This latter option can be carried out by interference RNA (RNAi) technology [12]. Among RNAi molecules, short-hairpin RNA (shRNA), also called expressed RNAi activator, is a plasmid-coded RNA. In contrast to other forms of RNAi [13,14], shRNA is continuously produced in the host cell and therefore induces a more lasting gene silencing effect. To be effective, shRNA must enter the cell and reach the nucleus, which is a major challenge to the design of a suitable delivery system. In fact, the success of gene therapy is strongly dependent on the development of safe and efficacious delivery systems that are able not only to reach the target cell, but also to provide an adequate intracellular disposition of the genetic material.

The cornea is a well-suited tissue for the application of gene-based therapies due to its easily accessible and immune-privileged status [15]. However, a historically poor transfection efficiency after topical instillation has hampered the development of gene delivery in the cornea, and the direct administration of the vectors into the corneal stroma is often used in preclinical studies. The design and evaluation of more efficient systems for nucleic acid delivery would promote the advance of gene therapy for corneal diseases, and the development of clinical trials, which are very scarce [16,17]. Most studies that evaluated gene delivery into the cornea have used viral vectors [18,19], which are well-established and effective, but apart from the technological drawbacks in terms of production and the limited size of the genetic material, they can induce oncogenicity and an immune response, mainly after repeated administrations [9]. Non-viral vectors offer a safer, more attractive alternative to viral vectors for corneal gene delivery due to their easy generation, ability to transport large nucleic acids or multiple genes, and more versatility for DNA conjugation. In this sense, our research group showed the potential of lipidic non-viral systems to efficiently transfect ocular tissues, including the corneal epithelium [20,21,22]. Lipid nanoparticles, which are at the forefront of the rapidly developing field of nanotechnology, present a high potential in biomedical application, such as the administration of chemotherapeutic drugs or nucleic acids [23,24]. Actually, solid lipid nanoparticles (SLNs) are regarded as one of the most effective non-viral vectors for gene therapy with additional advantages such as biocompatibility and the ease of large-scale production [25]. In addition, cationic SLNs present several advantages that make them appropriate for corneal gene therapy, since they interact with negatively charged mucus on the ocular surface after topical administration [26]. The mucoadhesive properties favor the retention time and, therefore, corneal permeation through endocytic uptake by the corneal epithelial cells.

Considering this situation, the aim of this work was to develop an SLN-based shRNA delivery system to downregulate MMP-9 in corneal cells using RNAi technology as a gene suppression therapy for the treatment of CNV-associated inflammation.

## 2. Materials and Methods

### 2.1. Materials

Precirol^®^ ATO 5 was a gift by Gattefossé (Madrid, Spain), DOTAP (1,2-dioleoyl-3-trimethylammonium-propane chloride salt) was purchased from Avanti Polar-lipids, Inc. (Alabaster, AL, USA), Tween 80 and dichloromethane were obtained from Panreac (Madrid, Spain). Nile Red, protamine sulfate salt Grade X (P), dextran (Mw of 3.26 KDa) (DX), deoxyribonuclease I (DNase I), sodium dodecyl sulfate (SDS), human insulin solution, and the Cell Counting Kit-8 (CCK-8) were obtained from Sigma-Aldrich (Madrid, Spain). The plasmid encoding a short-hairpin interference RNA against MMP-9 (p-shRNA-MMP-9; 4993 bp) and the plasmid encoding both a short-hairpin interference RNA against MMP-9 and the green fluorescent protein (GFP) (p-shRNA-MMP-9-GFP; 6675 bp) were obtained from GeneCopeia (Rockville, MD, USA). GelRed™ was obtained from Biotium (Fremont, CA, USA), and the materials used in electrophoresis on agarose gel were purchased from Bio-Rad (Madrid, Spain).

The human corneal epithelial (HCE-2) cell line and human umbilical vein endothelial cells (HUVEC) were supplied by American Type Culture Collection (ATCC, Manassas, VA, USA). For the cell culture, Dulbecco’s Modified Eagle’s Medium/Nutrient Mixture F-12 with Gluta MAX™ (DMEM/F-12 with GlutaMAX), Medium 200, Large vessel Endothelial Supplement (LVES), fetal bovine serum (FBS), and penicillin-streptomycin were acquired from Life Technologies (Thermo Fisher Scientific, Madrid, Spain). Epidermal growth factor (EGF) was obtained from Miltenyi Biotec (Madrid, Spain), and Trypsin-EDTA (ethylenediaminetetraacetic acid) from Lonza (Basel, Switzerland). Recombinant human tumor necrosis factor alpha (TNF-α) was obtained from Peprotech (London, UK), the Reporter Lysis Buffer (RLB) was purchased in Promega Biotech Iberica (Madrid, Spain), and the Micro BCA™ Protein Assay Kit and Geltrex^®^ LDEV-Free Reduced Growth Factor Basement Membrane Matrix from Thermo Scientific (Madrid, Spain).

Paraformaldehyde (PFA) was purchased from Panreac (Madrid, Spain) and phosphate buffered saline (PBS) and 4-(2-hydroxyethyl) piperazine-1-ethanesulfonic acid (HEPES) buffer was purchased from Gibco (Thermo Fisher Scientific, Madrid, Spain). Transfectin^®^ was acquired from Bio-Rad (Madrid, Spain). Enzyme-Linked Immuno Sorbent Assay (ELISA) for MMP-9 with the Duo Set Ancillary reagent kit was purchased from R&D Systems (Minneapolis, MN, USA).

The goat monoclonal anti-hMMP-9 antibody and Alexa Fluor 568-conjugated donkey anti-goat IgG were obtained from Abcam (Cambridge, MA, USA), and 4′,6-Diamidine-2′-phenylindole dihydrochloride (DAPI) Fluoromount-G^®^ was obtained from SouthernBiotech (Birmingham, AL, USA).

### 2.2. Methods

#### 2.2.1. Elaboration of the p-shRNA-MMP-9-Bearing Vectors

Different formulations based on SLN, dextran (DX), protamine (P), and plasmids (p-shRNA-MMP-9 or p-shRNA-MMP-9-GFP) were prepared as previously described [27]. First, SLNs were prepared with Precirol^®^ ATO 5 (Gattefossé, Madrid, Spain), DOTAP, and Tween 80, by using a solvent emulsification-evaporation technique. The final nanocarriers were formed by the addition of a complex previously prepared with P, p-shRNA-MMP-9, and DX. The vectors were formed with the following component ratios (*w*/*w*), expressed as DX/P/p-shRNA-MMP-9/SLN: 1/2/1/5, 1/1/1/5, 2/1/1/5 and 2/0.5/1/5.

#### 2.2.2. The Characterization of the Vectors

The plasmid, the complex of DX, P and the plasmid (component ratios (*w*/*w*) 2:1:1, respectively), and the final vector were subjected to electron microscopy negative staining for visualization. The vector suspension (10 µL) was adhered onto glow-discharged carbon coated grids for 60 s. The liquid remaining was removed by blotting on filter paper and the samples were stained with 2% uranyl acetate for 60 s. A JEOL JEM 1400 Plus transmission electron microscope (TEM, Tokyo, Japan) was used to obtain digital images.

The Correlation Spectroscopy (PCS) technique was used to measure the particle size and the polydispersity index and Laser Doppler Velocimetry (LDV) was used to measure the *Z* potential of the vectors. These measurements were carried out in a ZetaSizer Nano ZS (Malvern Panalytical, Malvern, UK). We analyzed the ability of the nanocarriers to bind the DNA, to protect it against DNase I digestion, and to release it by electrophoresis on 1% agarose gel containing Gel Red™ (30 min at 120 V). An Uvidoc D-55-LCD-20 M Auto transilluminator (Uvitec, Cambridge, UK) was used to analyze the gels. The vectors were diluted in MilliQ™ water (Merck Millipore, Madrid, Spain) up to a final concentration of 0.03 μg DNA/µL. To evaluate the capacity of the plasmid to be released, the vectors were treated with 4% sodium dodecyl sulfate (SDS) solution. For DNase I protection evaluation, a concentration of 1 U DNase I/2.5 μg DNA was added to the vectors and then the mixtures were incubated 30 min at 37 °C. Afterward, the p-shRNA-MMP-9 was released from the SLNs with SDS. An untreated 1-kb DNA ladder from NIPPON Genetics Europe (Dueren, Germany) was added as a molecular weight (MW) control.

#### 2.2.3. Cell Culture Conditions

The HCE-2 cell line was maintained in DMEM/F-12 GlutaMAX medium supplemented with 15% (*v*/*v*) heat-inactivated fetal bovine serum (FBS), insulin (4 mg/mL), EGF (10 ng/mL), and penicillin–streptomycin (1%). Cells were incubated with 5% CO_2_ at 37 °C and subcultured every 7 days using trypsin/EDTA. Cells from passages 1 to 4 were used to perform all in vitro assays.

#### 2.2.4. In Vitro Transfection Assays

For in vitro transfection, cells were seeded on 24-well plates at a density of 1 × 10^5^ cells/well and were allowed to adhere overnight. Cells were transfected with the vectors at a plasmid dose of 2.5 µg and maintained for 30 min at 37 °C and 5% CO_2_. Four hours after the addition of the vectors, the media were replaced, and the cell culture was allowed to grow up to 72 h at 37 °C and 5% CO_2_.

##### Percentage of Transfected Cells and GFP Production

To evaluate the rate of cells transfected, the vectors were prepared with the p-shRNA-MMP-9-GFP plasmid. The percentage of transfected cells was measured using a CytoFLEX (Beckman Coulter, Indianapolis, IN, USA) flow cytometer. Cells were rinsed with PBS (three times), separate from plates, and resuspended in PBS. Finally, samples were evaluated by flow cytometry at 525 nm collecting 1 × 10^4^ events per sample. The amount of intracellular GFP, in terms of relative fluorescence units (RFU)/mg total protein, was quantified by fluorometry. To do that, the culture media were substituted with 300 mL of 1× reported lysis buffer (RLB) and frozen. The cells were then scraped and centrifuged at 4 °C and 12,000× *g* for 2 min. The GFP fluorescence in the supernatant was measured by using a Glomax™ Multi-Detection System (Promega Biotech Iberica, Madrid, Spain). The fluorescence was corrected by the amount of protein, quantified by a Micro BCA™ Protein Assay kit (Thermo Scientific, Madrid, Spain).

##### Silencing of MMP-9

In order to know the capacity of the nanocarriers bearing the p-shRNA-MMP-9 plasmid to downregulate the MMP-9, we transfected HCE-2 cells using the same protocol described above. A p-shRNA-scramble (shRNAscr) plasmid and a naked p-shRNA-MMP-9 plasmid were used as negative controls. The silencing activity was evaluated at 72 h by measuring the MMP-9 secreted to the culture medium, and by detecting the MMP-9 at an intracellular level. The amount of MMP-9 secreted by the cells was measured in the culture medium by an ELISA kit (R&D Systems, Minneapolis, MN, USA).

The presence of MMP-9 in HCE-2 was evaluated by immunocytochemistry. For this purpose, untreated and transfected cells were seeded in 24-well plates with cover glasses in the bottom that were previously treated with the attachment factor protein (Thermo Fisher Scientific, Madrid, Spain). After adequate washing in PBS, the cells were blocked and permeabilized with PB buffer, 0.3% Triton X-100, 10% donkey serum for 30 min. A goat monoclonal anti-MMP-9 antibody in PBS buffer (2.5% donkey serum, 0.1% triton X-100) was then added and maintained for 1 h. After 3 more washes with PBS, the cells were dyed with the secondary antibody Alexa Fluor 568-conjugated donkey anti-goat IgG for 1 h in the dark. Finally, the nuclei were dyed with the mounting media DAPI Fluoromount-G^®^ (SouthernBiotech, Birmingham, AL, USA). The specificity of the staining was controlled by incubating cells without the primary antibody. The images were obtained with an inverted fluorescence microscope (Nikon TMS, Izasa Scientific, Madrid, Spain) at 40× magnification. Intracellular MMP-9 was also evaluated in HCE-2 cells previously stimulated with 10 ng of TNF-α, a proinflammatory mediator, for 6 h.

#### 2.2.5. Cell Viability

We measured the viability of the HCE-2 cells treated with the nanocarriers with the CCK-8 assay following the manufacturer’s protocol. In short, the cells were seeded in a 96-well plate at a density of 1 × 10^3^ cells/well and incubated overnight at 37 °C in a CO_2_ incubator followed by transfection with the different vectors. CCK-8 reactive solution was added to each well, incubated at 37 °C for 4 h. Finally, the absorbance was measured in a microplate reader at 450 nm.

#### 2.2.6. Cellular Uptake of Non-Viral Vectors

The internalization of the vectors by the HCE-2 cells was studied using a CytoFLEX flow cytometer. For this purpose, SLNs were labeled with the fluorescent Nile Red dye (λ = 590 nm) as previously described [28]. The cells were treated with the nanosystems for 2 h, resuspended in PBS and analyzed by flow cytometry at 610 nm, collecting 1 × 10^4^ events per sample.

#### 2.2.7. Intracellular Disposition of the Vectors

In order to know the intracellular disposition of the plasmid, cells were seeded at a density of 100,000 cells and 1 mL per well and incubated at 37 °C and 5% CO_2_ for 24 h in Millicell EZ slides (Merck Millipore, Madrid, Spain). Then, they were then treated with vectors containing the plasmid labeled with ethidium monoazide (EMA; Dro Byosystems S.L., San Sebastian, Spain). After 4, 12, and 24 h, the slides were rinsed with PBS and fixed with 4% PFA. Nuclei were labeled with DAPI Fluoromount-G^®^ (SouthernBiotech, Birmingham, AL, USA), and images were captured with the inverted fluorescence microscope (Nikon TMS, Izasa Scientific, Madrid, Spain)

#### 2.2.8. Cell Migration Assay

The effect of the formulations on the migration of HCE-2 cells was studied using a wound healing assay. For this purpose, HCE-2 cells were grown to confluence in 24-well plates coated with attachment factor containing gelatin (substrate of MMP-9) and a linear wound was created with a pipette tip. Afterward, cultures were rinsed twice with PBS to remove detached cells and 1 mL of fresh serum-free medium was added. Untreated cells, cells treated with the vector (corresponding to a plasmid dose of 2.5 µg), and cells stimulated with 10 ng of TNF-α were allowed to migrate at 37 °C under 5% CO_2_. After 4 h, the medium was changed by a 1 mL complete medium. The wound width was measured at 7 different points at 7, 24, and 48 h. The relative distance filled was calculated with the formula: *m* = (1 − *n_t_*/*r*) × 100%, where *m* is the migration, *n_t_* is the width of the scratch at time *t*, and *r* is the initial width of the scratch [29]. Blank nanoparticles were also assayed.

#### 2.2.9. HUVEC Tube Formation Assay

The HUVEC cell line was employed to evaluate whether MMP-9 downregulation is able to inhibit capillary tube formation. HUVEC cells were seeded onto Geltrex^®^ LDEV-Free Reduced Growth Factor Basement Membrane Matrix in 96-well plates and the culture medium was replaced with conditioned culture medium from HCE-2 cells untreated or treated with vector III, TNF-α, or TNF-α plus vector III. The plates were incubated at 37 °C, and tube formation was evaluated after 15 h of incubation under an optic inverted microscope. Morphometric measurements in captured images were obtained using the ImageJ software (National Institutes of Health, Bethesda, MA, USA) [30].

#### 2.2.10. Statistical Analysis

Significant differences between groups were analyzed with IBM SPSS Statistics for Windows, Version 23.0. (IBM Corp, Armonk, NY, USA). Normal distribution data were assessed by the Shapiro–Wilk test, and homogeneity of variance using the Levene test. The formulations were compared with the ANOVA test. Results are expressed as mean ± standard deviation and p values of less than 0.05 were regarded as statistically significant.

## 3. Results

### 3.1. Characterization

**Figure 1:**
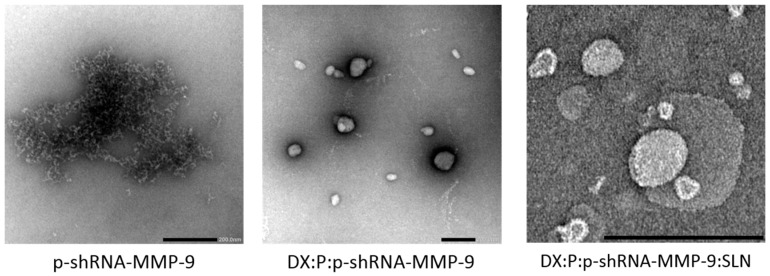
The images of p-shRNA-MMP-9, DX:P:p-shRNA-MMP-9 complex (*w*/*w* ratio 2:1:1), and DX:P:p-shRNA-MMP-9:SLN vector (*w*/*w* ratio 2:1:1:5), acquired by Transmission Electronic Microscopy (TEM). Scale bar: 200 nm. DX: dextran; P: protamine.

Figure 1 shows images of the p-shRNA-MMP-9 plasmid, the DX:P:p-shRNA-MMP-9 complex, and the DX:P:p-shRNA-MMP-9:SLN vector, acquired by TEM. We can observe that the plasmid, in dark, appears highly condensed after the binding to protamine and dextran. The picture also shows the spherical shape of the final vector.

The particle size of the vectors (Table 1) was in the range of nanometers, ranging from 182 to 216 nm, and all had a positive surface charge (+36.1 to +45.7 mV). Vector II presented a significantly higher particle size and a lower surface charge than the other vectors. The polydispersity index (PdI) was similar and under 0.30 in all cases, which indicates the homogeneity of the particle size.

**Figure 2:**
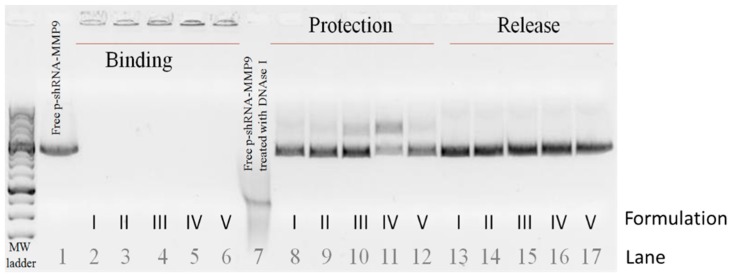
The agarose gel electrophoresis study of the capacity of the vectors to bind, protect, and release the p-shRNA-MMP-9. Lane 1: free p-shRNA-MMP-9; lanes 2 to 6: binding; lane 7: free p-shRNA-MMP-9 treated with DNase I; lanes 8 to 12: vectors treated with DNase I; lanes 13 to 17: vectors treated with SDS (induced release). Molecular weight (MW) ladder corresponds to the 1 kb DNA ladder from NIPPON Genetics Europe (Dueren, Germany).

To analyze the ability of the vectors to bind, protect, and release p-shRNA-MMP-9 plasmid, an agarose gel was conducted. The absence of bands in lanes 2–6 in Figure 2 indicates that all vectors were able to completely bind the p-shRNA-MMP-9. The gel also shows that formulations I to III (lanes 8–10, respectively) protected the plasmid more efficiently than formulations IV and V (lanes 11 and 12, respectively). After the treatment of the nanosystems with SDS, the plasmid was effectively released (lanes 13 to 17).

### 3.2. In Vitro Transfection and Cell Viability

**Figure 3:**
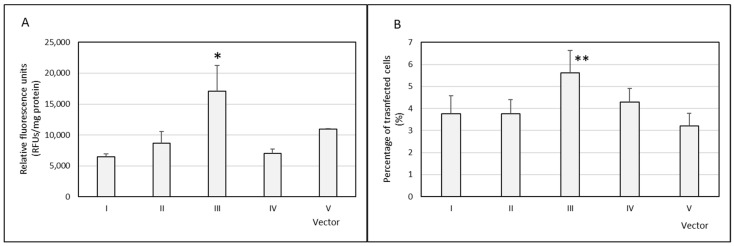
The transfection efficacy of the vectors in human corneal epithelial (HCE)-2 cells. (**A**) Relative fluorescence units per mg of protein (RFUs/mg protein) of the transfected cells. (**B**) Percentage of transfected cells (*n* = 4). Data are expressed as mean ± standard deviation. * *p* < 0.05 with respect to the other formulations. ** *p <* 0.05 with respect to formulations I, II, and V.

The percentage of HCE-2 transfected cells and the amount of GFP (expressed as RFUs corrected by the total amount of protein) were measured 72 h after the addition of vectors. The percentage of transfected cells ranged from 3.5% to 5.6%, and the vector that presented the highest transfection efficacy, expressed as both RFUs and percentage of cells transfected, was vector III (Figure 3). With all formulations, cell viability was always higher than 90%; no difference was detected compared to the untreated cells.

**Figure 4:**
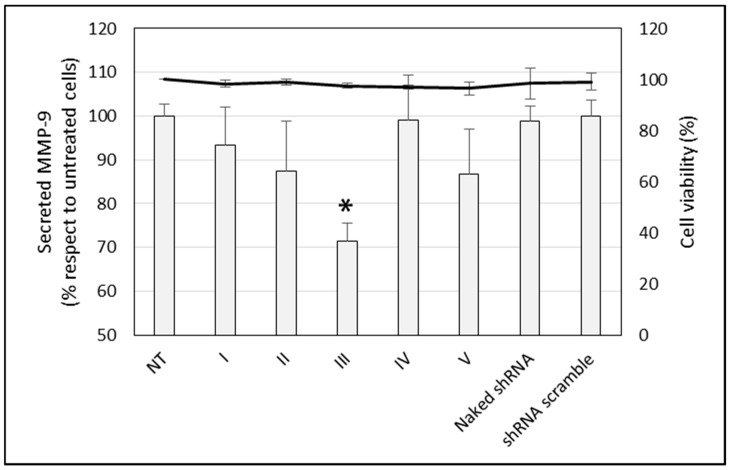
The secreted MMP-9 levels (bars, expressed as a percentage with respect to untreated cells) and viability (line) of HCE-2 cells treated with the vectors and naked p-shRNA-MMP-9. Data are expressed as mean ± SD of 4 experiments. NT: non-treated cells. * *p* < 0.05 with respect to NT cells.

To evaluate the silencing capacity of the nanoformulations, we quantified the MMP-9 secreted by the HCE-2 cells both untreated and treated with the vectors. Figure 4 shows that only vector III was able to significantly decrease the amount of MMP-9 secreted by the cells, with a silencing effect close to 30%. Cell viability was close to 100%, and no difference was detected compared to the untreated cells. Based on these results, vector III was selected for further experiments.

**Figure 5:**
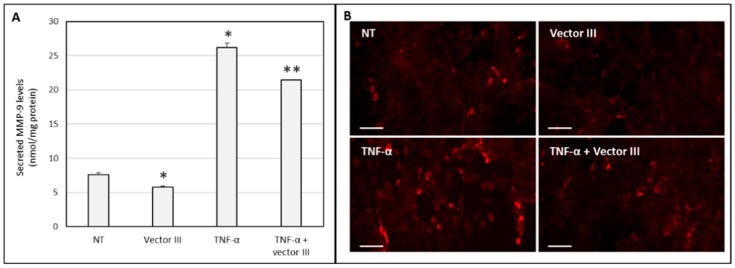
(**A**) The secreted MMP-9 levels in HCE-2 cells untreated and treated with vector III, TNF-α, or both. (**B**) Fluorescence images of immunostained MMP-9 in HCE-2 cells untreated and treated with vector III, TNF-α, or both. NT: non-treated cells. * *p* < 0.05 respect to non-treated cells (NT), ** *p* < 0.05 respect to TNF-α. Scale bar: 60 µm.

Figure 5A shows that the stimulation of HCE-2 cells with TNF-α significantly increased the levels of secreted MMP-9. The addition of vector III to non-stimulated and TNF-α-stimulated HCE-2 cells induced a decrease of about 20% in the secretion of MMP-9 levels to the culture medium. The intracellular expression of the metalloproteinase (Figure 5B) confirmed the silencing effect of the vector.

### 3.3. Cellular Uptake and Intracellular Trafficking

**Figure 6:**
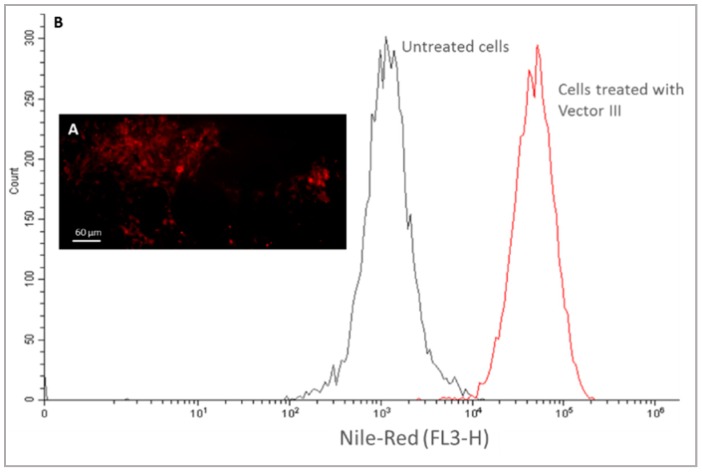
The uptake of the vector III in HCE-2 cells 2 h after transfection with Nile Red-labelled vector containing p-shRNA-MMP-9 against MMP-9. (**A**) Fluorescence microscopy image (40×); (**B**) flow cytometry histograms.

Flow cytometry histograms (Figure 6) show that vector III was efficiently internalized by the HCE-2 cells. A fluorescence microscopy photograph confirmed the high uptake degree of the nanosystems.

**Figure 7:**
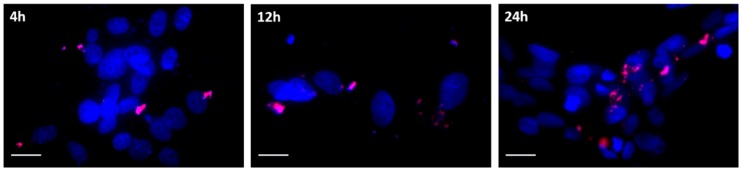
The fluorescence microscopy images of HCE-2 cells at different times after addition of vector III (4, 12, and 24 h). Ethidium monoazide (EMA)-labelled DNA (red) and nuclei stained with DAPI fluoromount-G^®^ (SouthernBiotech, Birmingham, AL, USA) (blue). Scale bar: 20 µm.

Figure 7 shows the fluorescence microscopy images captured at 4, 12, and 24 h post-addition of the vector III prepared with EMA-labelled DNA. As can be seen, the approach of the plasmid to the nucleus was increasing over time.

### 3.4. Migration Assay

**Figure 8:**
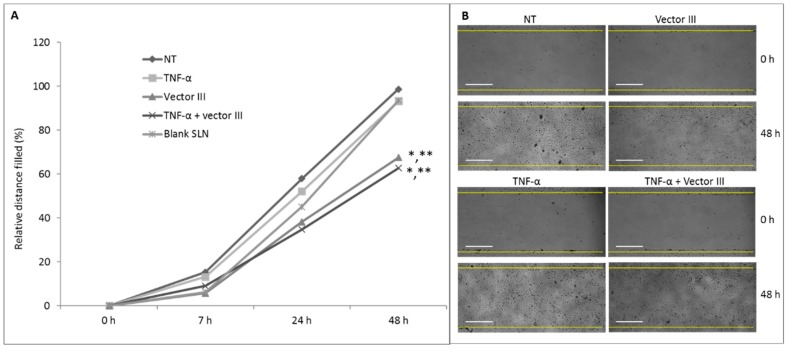
The effect of vector III on HCE-2 cell migration. (**A**) The evolution of the distance between cells of the edge of the wound; the mean data of reduction of the wound width from the 4 replicates (measures) in each condition. (**B**) Phase contrast representative images (4×). NT: non-treated cells. Statistics at 48 h. * *p* < 0.05 respect to NT, ** *p* < 0.05 respect to cells treated with TNF-α. Scale bar: 60 µm.

Figure 8 shows that vector III was able to reduce HCE-2 cell migration. While at 48 h, untreated cells reduced the wound width close to 100%, cells treated with vector III (stimulated and non-stimulated with TNF-α) produced a closure of only about 70%.

### 3.5. HUVEC Tube Formation Assay

**Figure 9:**
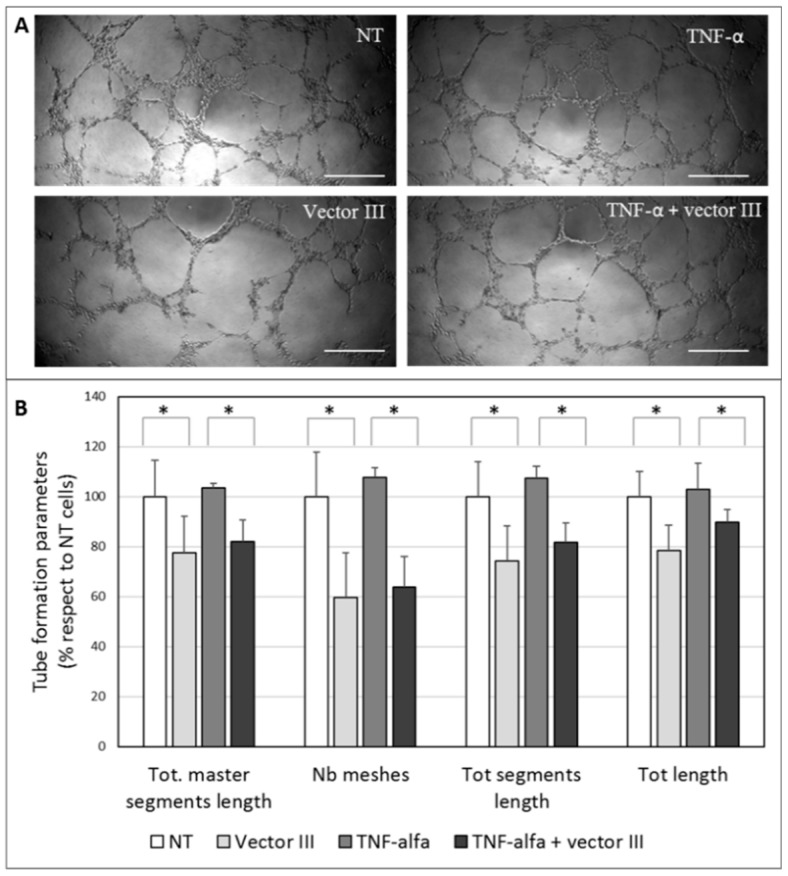
(**A**) The representative images obtained in the tube formation assay in human umbilical vein endothelial cells (HUVECs) and (**B**) the quantification of tube formation. Data were normalized relative to the values of non-treated cells (NT). * *p* < 0.05. Scale bar: 60 µm.

Figure 9 shows representative images of HUVEC cells treated with culture medium from HCE-2 untreated or treated with TNF-α, vector III, or TNF-α plus vector III. Both the photographs and the morphometric measurement show the inhibition of the tube formation, in terms of total master segment length, the number of meshes, total segment length, and total length (Figure 9B).

## 4. Discussion

Among the different strategies used in the treatment of CNV-associated inflammation, gene silencing has been shown to be useful for targeting proangiogenic factors. In a previous study [31], two plasmids encoding shRNA targeted against MMP-9 were effective to inhibit MMP-9 enzyme expression after intrastromal injection into the cornea of mice, stopping angiogenesis and decreasing the severity of herpetic keratitis. The authors concluded that the in vivo therapeutic application depends on the availability of efficient vehicles to deliver the RNAi to the target cells with low toxicity and in a method suitable for repeated administrations. In this regard, in the present work, we have developed vectors containing a shRNA against MMP-9 to be useful for the treatment of CNV. The systems are composed of SLNs, protamine, and dextran. The protamine has shown to increase the transfection efficacy of non-viral vectors due to its high ability to condense the genetic material, and therefore, to protect it from degradation, facilitate the entry into the nucleus, and to enhance transcription [32,33,34]. A polysaccharide, dextran, was also included because it is biocompatible and has demonstrated to contribute to nucleic acid delivery; moreover, it shows low cytotoxicity, is easily subject to chemical modification, have stealth properties, and is widely used for pharmaceutical and biomedical applications [35,36]. For the formation of the vectors, the complex dextran-protamine-plasmid is adsorbed on the nanoparticle surface, playing the electrostatic interactions between the components a fundamental role. The plasmid is highly condensed by the protamine and the cationic lipid, and dextran modulates these interactions, conditioning the final structure (Figure 1) and the physicochemical characteristics of the vectors.

All nanosystems that we prepared showed suitable features for transfection: particle size in the range of nanometers, and the ability to bind, release, and protect the p-shRNA-MMP-9 against nucleases. It is important to consider that the transfection success of a non-viral system depends on the equilibrium between the capacity to condense and release the DNA [37]. This balance is conditioned by the nature and proportion of the components in the vector, which bind the genetic material by electrostatic interactions, as mentioned above. Accordingly, vector IV, which was prepared with the lowest proportion of protamine, provided the lowest protection against DNase I.

All vectors were able to transfect HCE-2 cells, with vector III being the most efficacious in terms of percentage of cells transfected and the amount of protein expressed as well. The higher efficacy of vector III was confirmed when we measured the protein MMP-9, secreted by the HCE-2 cells. This vector was able to induce a decrease of approximately 30% in the amount of MMP-9 protein synthesized by the cells. The effect of the vector is related to extensive internalization and to the ability of the plasmid to be released in the cytoplasm close to the nuclear membrane, as Figure 6 and Figure 7 show.

Once we demonstrated the efficacy in silencing MMP-9 in HCE-2 cells, an SLN-based vector was evaluated in TNF-α stimulated cells. TNF-α is a proinflammatory mediator that plays an important role in a variety of corneal diseases [38]. It disrupts the barrier function of human corneal epithelial cells and contributes to ocular inflammation [39,40]. TNF-α is reportedly elevated in corneas from individuals suffering keratitis [40], and this cytokine has been shown to stimulate MMP-9 activity in HCE cells [6]. We confirmed the increase in MMP-9 levels after the stimulation of the HCE-2 cells with TNF-α and the ability of the SLN-based vector to reduce the production of the MMP-9 in TNF-α-induced cells. These results indicate the suitability of TNF-α induced HCE-2 cells as an in vitro model to evaluate new formulations based on the MMP-9 downregulation for CNV.

MMPs are enzymes that are capable of cleaving numerous extracellular matrix proteins, which facilitates the migration of corneal epithelial cells to the underlying stroma [41,42,43,44]. Actually, the increased production and activity of MMPs are related to a more migratory and invasive cell phenotype [45,46]; conversely, the reduction of MMP-9 in HCE cells inhibits cell migration [47]. The suppression of the production of MMP-9 in the ocular surface contributed to the improvement in the corneal barrier function in a model of dry eye mice [48]. In our study, vector III was able to decrease the migration of HCE-2 cells. This effect can be related to the inhibition of MMP-9 production. MMP-9 degrades type IV collagen and gelatin substrates [49], and, therefore, a decrease in MMP-9 levels will result in a lower capacity to degrade the gelatin, used in this study as extracellular matrix protein. This strategy may be clinically helpful for the treatment of corneal diseases in which MMP-9 activity and inflammatory cytokines are upregulated.

As mentioned above, MMP-9 has also been revealed to play an important role in angiogenesis and, specifically, in angiogenesis associated with herpetic keratitis [50]. Corneal avascularity relies on the balance between proangiogenic and antiangiogenic factors. When the counterbalance between both kinds of factors shifts toward proangiogenic factors, new blood vessels grow from pre-existing vasculature at the pericorneal plexus [51]. In response to a stimulus such as an injury, the corneal epithelial cells release angiogenic growth factors that bind to receptors on the vascular endothelial cells of pericorneal vessels [9], and although CNV occurs in the corneal stroma, it is regulated by corneal epithelium-expressed factors. Vector III was able to partially suppress the tube formation in an in vitro HUVEC tube formation assay. Morphometric measurements revealed that total master segment length, the number of meshes, total segments length, and total length decreased when the HUVEC cells were supplemented with the conditioned culture medium from HCE-2 cells treated with the vector. The magnitude of the inhibition of the tube formation was in the same order as the reduction of the secreted MMP-9 levels by the HCE-2 cells. The effect of our vector was observed also for TNF-α-stimulated cells. We previously showed, in rabbit corneal explants, the ability of SLN to transfect different layers of the cornea [20]. Therefore, we expect that after the in vivo topical administration of the vector, MMP-9 downregulation could have effects not only in epithelial cells, but also in stromal keratocytes and even vascular endothelial cells, which are also involved in CNV, contributing to the total effect.

In conclusion, we demonstrated the ability of non-viral vectors based on SLN to downregulate MMP-9 expression in HCE-2 cells by gene silencing and, consequently, to inhibit cell migration and tube formation. These results highlight the potential of lipid nanoparticles as gene delivery systems for the treatment of CNV-associated inflammation by RNAi technology. The next step to assess the potential of this new vector will be to move towards in vivo studies.

## 5. Patents

Nanoparticles for gene therapy: EP2460516 (A2), EP2460516 (A4), EP2460516 (B1), ES2351756 (A1), ES2351756 (B1), US2012183589 (A1), US9675710 (B2), WO2011015701 (A2), WO2011015701 (A3).

Lipid Nanoparticles for Treating Ocular Diseases: EP2656837, (A1), EP2656837 (A4), ES2385080 (A1), ES2385080 (B1), WO2012085318 (A1).

## Figures and Tables

**Table 1:** The characterization of nanovectors bearing the p-shRNA-MMP-9 plasmid. Size (nm), Zeta potential (mV), and polydispersity index are presented as mean ± SD (*n* = 3).

Vector	DX:P:p-shRNA-MMP-9:SLN Ratio	Size (nm)	ZP (mV)	PdI
I	1:2:1:5	186 ± 5	+40.7 ± 1.0 **	0.21 ± 0.01
II	1:1:1:5	216 ± 4 *	+36.1 ± 1.0 *	0.28 ± 0.02
III	2:1:1:5	190 ± 3	+45.1 ± 1.2	0.22 ± 0.01
IV	2:0.5:1:5	189 ± 4	+42.8 ± 0.8 **	0.21 ± 0.01
V	3:1:1:5	182 ± 2	+45.7 ± 1.4	0.23 ± 0.01

* *p* < 0.05 with respect to the other formulations. ** *p* < 0.05 with respect to formulations II, III, and V. Abbreviations: DX: dextran; SLN: solid lipid nanoparticle; MMP: metalloproteinase; P: protamine; ZP: zeta potential; PdI: polydispersity index.
